# ADAM: automated data management for research datasets

**DOI:** 10.1093/bioinformatics/bts644

**Published:** 2012-10-29

**Authors:** Mark Woodbridge, Christopher D. Tomlinson, Sarah A. Butcher

**Affiliations:** Centre for Integrative Systems Biology and Bioinformatics, Imperial College London, London SW7 2AZ, UK

## Abstract

Existing repositories for experimental datasets typically capture snapshots of data acquired using a single experimental technique and often require manual population and continual curation. We present a storage system for heterogeneous research data that performs dynamic automated indexing to provide powerful search, discovery and collaboration features without the restrictions of a structured repository. ADAM is able to index many commonly used file formats generated by laboratory assays and therefore offers specific advantages to the experimental biology community. However, it is not domain specific and can promote sharing and re-use of working data across scientific disciplines.

**Availability and implementation:** ADAM is implemented using Java and supported on Linux. It is open source under the GNU General Public License v3.0. Installation instructions, binary code, a demo system and virtual machine image and are available at http://www.imperial.ac.uk/bioinfsupport/resources/software/adam.

**Contact:** m.woodbridge@imperial.ac.uk

## 1 INTRODUCTION

Research studies often require the collation of large quantities of working data of varied types, typically stored in the file system of a researcher’s computer. Use of such an unstructured repository has advantages over specialized data silos or a generic document management system in terms of familiarity, support for heterogeneous file types and ease of data modification. Further benefits such as data security, scalability and remote access can be gained by using a centrally managed networked file system.

However, as data volumes increase over the lifetime of a project, it can be increasingly hard to track the origin, status and location of datasets in such systems. The use of private repositories in collaborative projects does not encourage data sharing, meaning that files are often unnecessarily duplicated. Standard file systems also do not provide any intelligence to project coordinators or infrastructure providers as to the nature of data being acquired.

ADAM is a software tool designed to address these issues and has been developed in consultation with researchers involved in cross-discipline systems biology studies. It is designed to automatically classify, annotate and index data files without disrupting a user’s typical workflow. The researcher can choose to consult the additional metadata when necessary to retrieve or share data items and can provide further annotation as desired. As a centralized system, it can enable data sharing, assist researchers in finding potential collaborators and can provide aggregated statistics indicating data type prevalence and instrument usage amongst others.

## 2 USAGE

ADAM is a storage system that acts as a networked fileserver, accessible using the SMB protocol by any computer running a recent version of Mac OS, Windows and Linux. Any data files stored in the system are automatically inspected and indexed, regardless of the file system directory structure. This is an approach shared with the ‘dropbox’ component of OMERO (Allan *et al.*, 2012) for image archival. Files of unrecognized type are added to the index but are not associated with any additional metadata until it is optionally provided by the user. Table 1 lists the recognized file types and the typical metadata automatically extracted for each type.

**Table 1.** Supported file formats

Type	Supported formats	Example metadata
Publication	Journal article PDF	Author, journal, DOI, date
Document	Word, Excel, PowerPoint	Word count, named entities
Script	Python, MATLAB, R	Language, line count
Image	JPEG, GIF, Zeiss LSM, Leica LIF, SimplePCI CXD^a^	Image count, dimensions
Dataset	Affymetrix CEL, Waters MS, Bruker NMR	Acquisition parameters

^a^Formats list additionally includes those accessible using the Bio-Formats library.

A web interface viewable using any modern browser provides access to data, metadata and tools for browsing, filtering and searching. Data can be ordered by upload or modification date, providing a chronological view of acquisition. Uploaded journal articles are indexed automatically using heuristics to extract biographic information, and can be browsed using facets such as author, publication date and Medical Subject Headings terms. Filtering is possible based on file name, type, tag and date of upload or modification. Searching allows data to be identified using these filter criteria in addition to an index of the text extracted from relevant file types. This enables queries such as ‘Find all Word and Excel documents that mention NFKB1’ and ‘Find mass spectrometry data from the Aspergillus project that were generated last year’.

The web interface allows users to preview documents and images, to tag data items with textual annotations or ontology terms and to upload files when direct access to the fileserver is not possible. It also attempts to incorporate knowledge from public sources where appropriate, for example, linking out to related articles in PubMed or UniProt when a protein name is recognized in an uploaded document. 2D barcodes are generated for uploaded documents such as experimental protocols so that their content can easily be transferred to mobile devices for consultation in the laboratory.

Data can easily be shared with individual users or administrator-defined user groups, typically corresponding to project membership. The recipients are notified in real time over the web and can optionally be notified through email when sharing is configured. If a folder is shared, then any files subsequently added to that dataset will automatically be made available to the recipients. Files or folders can also be published, producing a URL that can be distributed to users without an account on the system. This link can be revoked at any time, thus enabling ad hoc data sharing with minimal effort.

Importantly, the use of the web interface is entirely optional and no manual steps are required to import data into the ADAM repository beyond copying files to the server. The interface can be used whenever a user wishes to take advantage of the features that the system provides over and above a traditional networked drive.

In addition to the features designed for end users, there are also clear benefits to infrastructure providers in having access to aggregated resource of data and the associated systematically derived metadata. It enables, for example, the determination of the most widely used file formats, and, by deduction, the kinds of experimental techniques and high-throughput assays being performed, and which instruments were used for acquisition. This should enable better planning including targeted use of hardware resources and development of analysis software to satisfy demand. It would also allow, with user consent, for alerts to be provided if researchers have uploaded ‘similar’ datasets or articles as a means to initiate collaboration.

## 3 IMPLEMENTATION

ADAM consists of several loosely coupled components:

- A network-accessible file system (SMB using Samba).
- File system monitoring daemon written using Java/inotify.
- Metadata database stored in the MongoDB system.
- Full-text index provided using the ElasticSearch library.
- JSON HTTP API written using the Java/Play Framework.
- Web interface implemented with HTML/CSS/Javascript.

The principal components are the data store, which will typically be located on a disk array mounted on the ADAM server and the metadata store, which is implemented using a high-performance document-oriented database well suited to ad hoc schema changes. All indexed files are assigned a globally unique identifier so that they can be unambiguously linked to their metadata and referenced externally even if their location changes.

Data type classification, metadata extraction and format conversion are performed using the Apache Tika, LOCI Bio-Formats (Linkert *et al.*, 2010) and Apache PDFBox libraries. The system additionally attempts to perform named-entity recognition using the Whatizit (http://www.ebi.ac.uk/webservices/whatizit/) or Reflect (O’Donoghue *et al.*, 2010) web services and generates repository content statistics using MongoDB MapReduce queries, which it makes viewable on the web to administrators.

The ADAM system architecture is modular by design, with a decoupled user interface. The HTTP API provides access to all search and retrieval functionality, meaning that custom interfaces can easily be developed to supplement the provided web application. The file type detection module of the server consists of pluggable components, making it straightforward for an experienced developer to extend the system to support new types. Simple HTML view templates allow the web interface to be customized to display these types.

The simplest deployment strategy for ADAM involves instantiating a provided VirtualBox machine image that bundles the core components with a CentOS Linux installation and includes scripts to start/stop the server and to add new users. The only required post-installation step is to configure any user-facing firewall and to mount a remote drive to act as the data store, if required.

## 4 DISCUSSION

ADAM is designed to encourage the usage of centralized storage in preference to potentially unreliable local facilities. It achieves this by providing access to valuable additional features without restricting researchers in how they organize their working data. It is not an alternative to established public repositories for publishing data associated with peer-reviewed research, nor does it eliminate the need for the types of manual annotation that facilitate data integration. It does, however, assist researchers in dealing with increasingly overwhelming digital data volumes by providing a powerful search interface and promotes data re-use by making it easy to share datasets with collaborators long before publication. It can additionally provide administrators with a means to analyse the nature and quantity of data being generated by users of the system and so ultimately benefit researchers and their supervisors too.
